# Consequences of *Zmat3* loss in *c-MYC*- and mutant *KRAS*-driven tumorigenesis

**DOI:** 10.1038/s41419-020-03066-9

**Published:** 2020-10-20

**Authors:** Sarah A. Best, Cassandra J. Vandenberg, Etna Abad, Lachlan Whitehead, Laia Guiu, Sheryl Ding, Margs S. Brennan, Andreas Strasser, Marco J. Herold, Kate D. Sutherland, Ana Janic

**Affiliations:** 1 The Walter and Eliza Hall Institute of Medical Research, 1G Royal Parade, Parkville, Melbourne, VIC 3052 Australia; 2 Department of Medical Biology, University of Melbourne, Parkville, Melbourne, VIC 3052 Australia; 3 Department of Experimental and Health Sciences, Universitat Pompeu Fabra, Doctor Aiguader 88, 08003 Barcelona, Spain

**Keywords:** Cancer, Cancer models, Cell biology, Mechanisms of disease

## Abstract

TP53 is a critical tumor suppressor that is mutated in approximately 50% of human cancers. Unveiling the downstream target genes of TP53 that fulfill its tumor suppressor function is an area of intense investigation. *Zmat3* (also known as *Wig-1* or *PAG608*) is one such downstream target of p53, whose loss in hemopoietic stem cells lacking the apoptosis and cell cycle regulators, Puma and p21, respectively, promotes the development of leukemia. The function of Zmat3 in tumorigenesis however remains unclear. Here, to investigate which oncogenic drivers co-operate with *Zmat3* loss to promote neoplastic transformation, we utilized *Zmat3* knockout mice in models of *c-MYC*-driven lymphomagenesis and *Kras*^G12D^-driven lung adenocarcinoma development. Interestingly, unlike loss of *p53*, *Zmat3* germline loss had little impact on the rate of tumor development or severity of malignant disease upon either the *c-MYC* or *Kras*^G12D^ oncogenic activation. Furthermore, loss of *Zmat3* failed to rescue *Kras*^G12D^ primary lung tumor cells from oncogene-induced senescence. Taken together, we conclude that in the context of *c-MYC-*driven lymphomagenesis or mutant *Kras*^G12D^-driven lung adenocarcinoma development, additional co-occurring mutations are required to resolve Zmat3 tumor suppressive activity.

## Introduction

P53 is a pivotal suppressor of cancer development and progression. The importance of p53 tumor suppression function and response to anti-cancer agents has driven the development of novel strategies to target the p53 pathway in cancer therapy^1^. Although the role of p53 in preventing tumor development has provoked intense investigation, the underlying mechanisms crucial in tumor suppression downstream of p53 have remained elusive^2–4^. Cell cycle arrest, cell senescence and apoptosis have been implicated in p53-mediated tumor suppression^2,5,6^. However, no spontaneous tumors arise in mice lacking *Puma*, *Noxa*, and *p21*, the critical mediators of p53-induced apoptosis and G1/S cell cycle arrest^7^. Likewise, in several mouse tumor models in different cellular compartments, it has been demonstrated that p53 mutants defective in the induction of apoptosis, cell cycle arrest and cell senescence retain tumor-suppressor activity^8–10^. Collectively, these studies demonstrate that p53 must suppress tumor development through currently underappreciated processes that act apart from, or in addition to, the induction of apoptosis, cell cycle arrest and cell senescence^7,11–13^.

Utilizing an in vivo shRNA screening approach, we recently demonstrated that *Zmat3* acts as a tumor suppressor gene downstream of p53, with its loss cooperating with inactivation of cell cycle arrest, cell senescence and apoptosis inducers (PUMA and p21) in the development of hematopoietic malignancy^14^. Zmat3 has been described as an RNA-binding zinc-finger protein that is involved in post-transcriptional regulation of gene expression and as such is found expressed in a broad range of tissues^15^. Moreover, akin to being a p53 target gene, Zmat3 has been reported to exert *bona fide* roles in the control of cell proliferation and cell survival^16^, where it acts through the regulation of *p53* and *p21* mRNA^17–20^. Interestingly, while both decreased and increased *Zmat3* mRNA expression have been reported in human tumors^15^ the exact role of Zmat3 in tumorigenesis remains elusive. We therefore hypothesized that the function of Zmat3 in tumorigenesis may be context dependent, influenced by genetic background, cell type or oncogenic drivers specific to each tumor. To further investigate the functions of Zmat3 in tumor suppression, we evaluated the impact of its loss in c-MYC-driven lymphomagenesis and mutant *Kras*^G12D^-driven non-small cell lung cancer (NSCLC)^21^, in which the p53 pathway has been shown to play a critical tumor suppressive role^22–24^.

## Materials and methods

### Mice

Animal experiments were conducted according to the regulatory standards approved by the Animal Ethics Committees of the Walter and Eliza Hall Institute of Medical Research (WEHI) and the Barcelona Biomedical Research Park (PRBB). *E*µ-*Myc*^24^, *p53*^25,26^*, Kras*^LSL-G12D/+^ mice^21^ and *Zmat3*^14^ mice have been previously described. All animals were maintained on a C57BL/6 background, and equal proportions of males and females were used in all experiments. Seven- to eight- week-old *Kras*^LSL-G12D/+^ compound mice were intranasally (i.n.) infected with 20 μL of 1 × 10^10^ PFU/mL Ad5-CMV-Cre virus (University of Iowa Gene Transfer Core Facility) according to standard procedures^27^. Lungs were harvested at defined time points (6-, 10- and 16-weeks post i.n. infection) or when mice showed signs of morbidity for Kaplan-Meier survival analysis.

### qRT-PCR analysis

Pre-B cells were treated with 5 Gy γ-irradiation or with 10 µM Nutlin3a (Nut3a), in the presence of 25 µM QVD-OPH (Sigma-Aldrich, St. Louis, MO, USA) in culture. WT and *Zmat3*^−/−^ mice, 2–5 months old, were either exposed to 8 Gy γ-irradiation or left untreated and lung tissue was harvested after 6 h. RNA was extracted using Trizol reagent (Invitrogen, Carlsbad, CA, USA) and converted to cDNA using the SuperScript First Strand Synthesis Kit (Thermo Fisher Scientific). Quantitative RT-PCR was performed using TaqMan probes (Thermo Fisher Scientific) for *Zmat3* (Mm01292424_m1), *Puma* (Mm00519268_m1), *p21* (Mm00432448_m1) and analyzed by the ΔΔCT method relative to *HMBS* (Mm01143545_m1).

### Flow cytometry

B lymphoid cells were isolated from bone marrow collected from both femurs and tibias of 4 week-old *Eµ-Myc* mice and stained with the following antibodies: B220-PE (clone RA3-6B2), IgM-FITC (clone 5.1) and IgD-FITC (clone 11-26C). Viable pre-B cells (B220^+^IgM^-^IgD^-^PI^-^) were isolated by flow cytometry using an ARIA flow cytometer (Becton Dickinson, San Jose, CA, USA). 1 × 10^6^ cells/mL were cultured in DMEM (Thermo Fisher Scientific, Waltham, MA, USA), 10% FBS (Sigma-Aldrich), 100 U/mL penicillin (Thermo Fisher Scientific), 100 µg/mL streptomycin (Sigma-Aldrich), 50 μM 2-Mercaptoethanol and 100 μM asparagine (Thermo Fisher Scientific) during the time-course. Antibodies were produced in-house.

Immunophenotyping of lymphomas was performed on single-cell suspensions stained with surface-marker-specific antibodies: CD43-BV650 (BD Biosciences; #740464; dilution 1:800); B220-APC-Cy7 (BD Biosciences; #552094; dilution 1:200); IgD-BV510 (BD Biosciences; #563110; dilution 1:200); IgM-FITC (BD Biosciences; #553437; dilution 1:100) to define pro/pre B cells (B220^+^CD43^+^) and Ig+ B cells (IgM and IgD). Samples were run on the LSR II (BD Biosciences). Dead cells (PI^+^) were excluded from analysis.

For immune cell analysis of lung tissue, the left lobe of each lung was weighed and harvested in PBS for digestion to generate a single cell suspension and blocked as described previously^28^. Blocked cells were incubated with fluorochrome-conjugated antibodies to define key immune cell populations from the lymphoid and myeloid compartments: B cells (CD45^+^CD3^−^CD19^+^), T cells (CD45^+^CD3^+^), NK cells (CD45^+^CD3^−^NKp46^+^DX5^+^), alveolar macrophages (CD45^+^CD11c^+^CD103^−^), CD103 dendritic cells (CD45^+^CD11c^+^CD103^+^), monocytes (CD45^+^CD11b^+^Ly6G^−^SSC^lo^), neutrophils (CD45^+^CD11b^+^Ly6G^+^) and eosinophils (CD45^+^CD11b^+^Ly6G^−^SSC^hi^) as described previously^29^.

### Histology and immunohistochemistry

Lungs were perfused and fixed with 4% paraformaldehyde (Sigma-Aldrich) for 24 h at 4 °C and embedded in paraffin. Sections 2 μm thick were stained with hematoxylin and eosin (H&E), as described previously^28^. Immunohistochemistry was performed as previously described^28^, using antibodies against Ki67 (Cell Signaling, Beverly, MA, USA; #12202), Nkx2.1/TTF-1 (Dako/Agilent Technologies, Santa Clara, CA, USA; #M3575) and Hmga2 (BioCheck, Foster City, CA, USA; #59170AP). H&E stained lung sections were imaged at low power and abnormal regions were masked on Image J software (Softonic International, Barcelona, Spain). Abnormal regions were classified as hyperplastic or adenoma/adenocarcinoma (ADC) based on morphology, and lesion number per lung was quantified. Hmga2 and Ki67 stained images were scored automatically using a custom Image J^30^ pipeline which utilized the Stardist plugin for nuclear segmentation^31^. Nuclei were then scored based on their intensity in the brown channel after color deconvolution. Tumor burden was determined using Image J through a combination of rank and morphology filters, followed by an auto-threshold to select the whole tissue, and the regions of dense nuclei comprising the tumor.

### Cell growth analysis

Cell lines were seeded from a single cell suspension derived from the left lobe of the lung. At each passage, cells were counted and 2 × 10^5^ cells were re-seeded into one well of a 6-well plate in primary cell medium (DMEM/F-12+GlutaMAX (Thermo Fisher Scientific), 10% FBS (Sigma-Aldrich), 100 U/mL penicillin (Thermo Fisher Scientific), 100 μg/mL streptomycin (Thermo Fisher Scientific), 0.04 mg/mL hydrocortisone (Sigma-Aldrich), 1× Insulin-Transferrin-Selenium-Ethanolamine (ITS-X; Thermo Fisher Scientific) and 5 ng/mL epidermal growth factor (Sigma-Aldrich)).

### Statistical analysis

Prism (Version 8; GraphPad) software was used for all statistical analyses. Two-group comparisons were made using two-tailed Student’s *t-*tests assuming equal variances and multivariate comparisons were performed using one-way ANOVA (Kruskal–Wallis test) with Tukey’s multiple comparisons. Animal survival data were plotted using Kaplan–Meier curves and compared using log-rank (Mantel–Cox) test.

## Results and discussion

### Loss of Zmat3 does not accelerate Myc driven lymphoma development

**Fig. 1:**
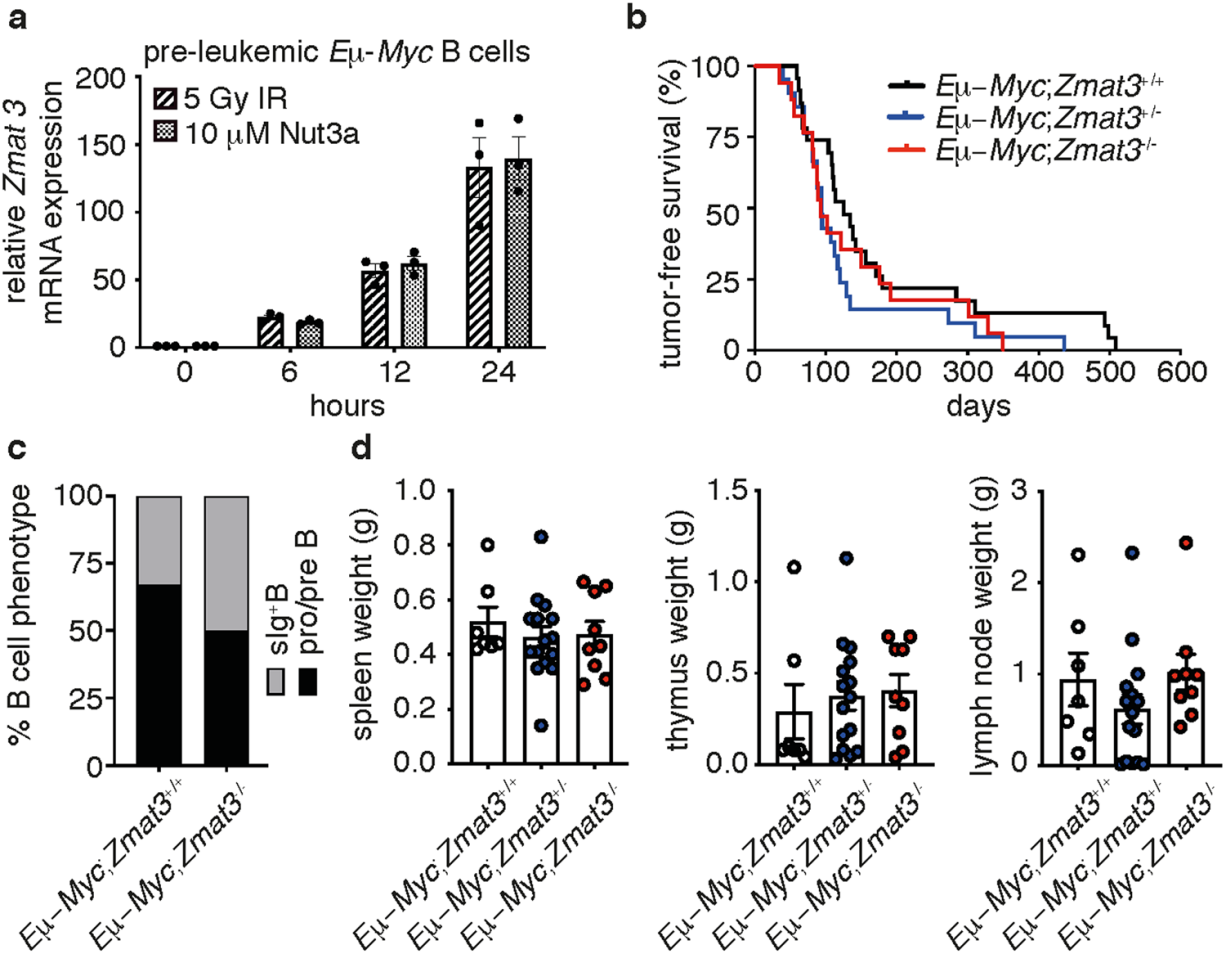
Loss of Zmat3 has no impact on lymphoma development in *Eu-Myc* mice. **a** *Zmat3* mRNA expression in pre-leukemic *Eµ-Myc* B lymphoid cells 0, 6, 12 and 24 h following 5 Gy γ-irradiation (IR) or treatment with 10 µM Nutlin3a (Nut3a). Mean ± SEM. **b** Lymphoma-free survival of *Eµ-Myc* (*n* = 23), *Eµ-Myc*;*Zmat3*^+/^^−^ (*n* = 21) and *Eµ-Myc*;*Zmat3*^−/^^−^ (*n* = 17) mice. Median lymphoma-free survival: *Eµ-Myc*: 125 days; *Eµ-Myc*;*Zmat3*^+/−^: 94 days; *Eµ-Myc*;*Zmat3*^−/^^−^: 93 days. *Eµ-Myc vs Eµ-Myc*;*Zmat3*^+/^^−^ *p* = 0.0718; *Eµ-Myc vs Eµ-Myc*;*Zmat3*^−/^^−^ *p* = 0.3231. **c** The proportions of Ig^+^ B cell lymphomas and pro-B/pre-B cell lymphomas in sick *Eµ*-*Myc*;*Zmat3*^−/−^ (*n* = 4) and sick *Eµ*-*Myc* (*n* = 3) mice. No significant differences were observed; *p* = 0.5 calculated by two-tailed *t*-test. **d** Spleen, thymus and lymph node weight (grams) in sick *Eµ-Myc* (*n* = 7), *Eµ-Myc*;*Zmat3*^+/^^−^ (*n* = 15) and *Eµ-Myc*;*Zmat3*^−/^^−^ (*n* = 9) mice at ethical endpoint. Mean ± SEM.

To interrogate the role of Zmat3 in tumorigenesis in vivo, we investigated the collaboration of loss of *Zmat3* with deregulated expression of the oncogene *c-Myc*, using the *Eµ-Myc* model of pre-B/B lymphoma development^24^. Critically, *Eμ-Myc* mice have been extensively used to study the defects in the p53-pathway (e.g. impact of loss of *Puma* or loss of one allele of *p53*) on tumorigenesis^22,24,32^. First, we examined the expression of *Zmat3* in pre-leukemic *Eµ-Myc* pre-B lymphoid cells isolated from the bone marrow of 4-week old *Eµ-Myc* mice, with or without activation of p53. Indeed, *Zmat3* mRNA expression was induced in pre-leukemic pre-B lymphoid cells in response to both the MDM2 inhibitor Nutlin3a^33^ and γ-irradiation (Fig. 1a), confirming that *Zmat3* is a *bona fide* p53 target gene. It has been shown that loss of one allele of *p53* significantly accelerates lymphoma development in *Eμ-Myc* mice^3,11,24^. Therefore, we next investigated whether loss of Zmat3 would accelerate lymphoma development in *Eµ-Myc* mice. To address this question, *Zmat3* knockout (*Zmat3*^−/−^) mice^14^ were crossed with *Eµ-Myc* transgenic mice^24^ and monitored for lymphoma development. Strikingly, neither loss of one allele or even both alleles of *Zmat3* altered the onset of lymphoma in *Eµ-Myc* mice (Fig. 1b). Post-mortem analysis demonstrated no significant difference in leukemic burden, the overall proportion of pre-B *vs* mature B-cell tumors, or in the weight of hematopoietic organs between sick *Eµ-Myc;Zmat3*^*−/−*^ *vs* *Eµ-Myc* mice (Fig. 1c, d). Taken together, these results reveal that, unlike loss of *p53*, germline loss of *Zmat3* fails to accelerate c-MYC-driven lymphoma development.

### The impact of Zmat3 loss on adult lung development

**Fig. 2:**
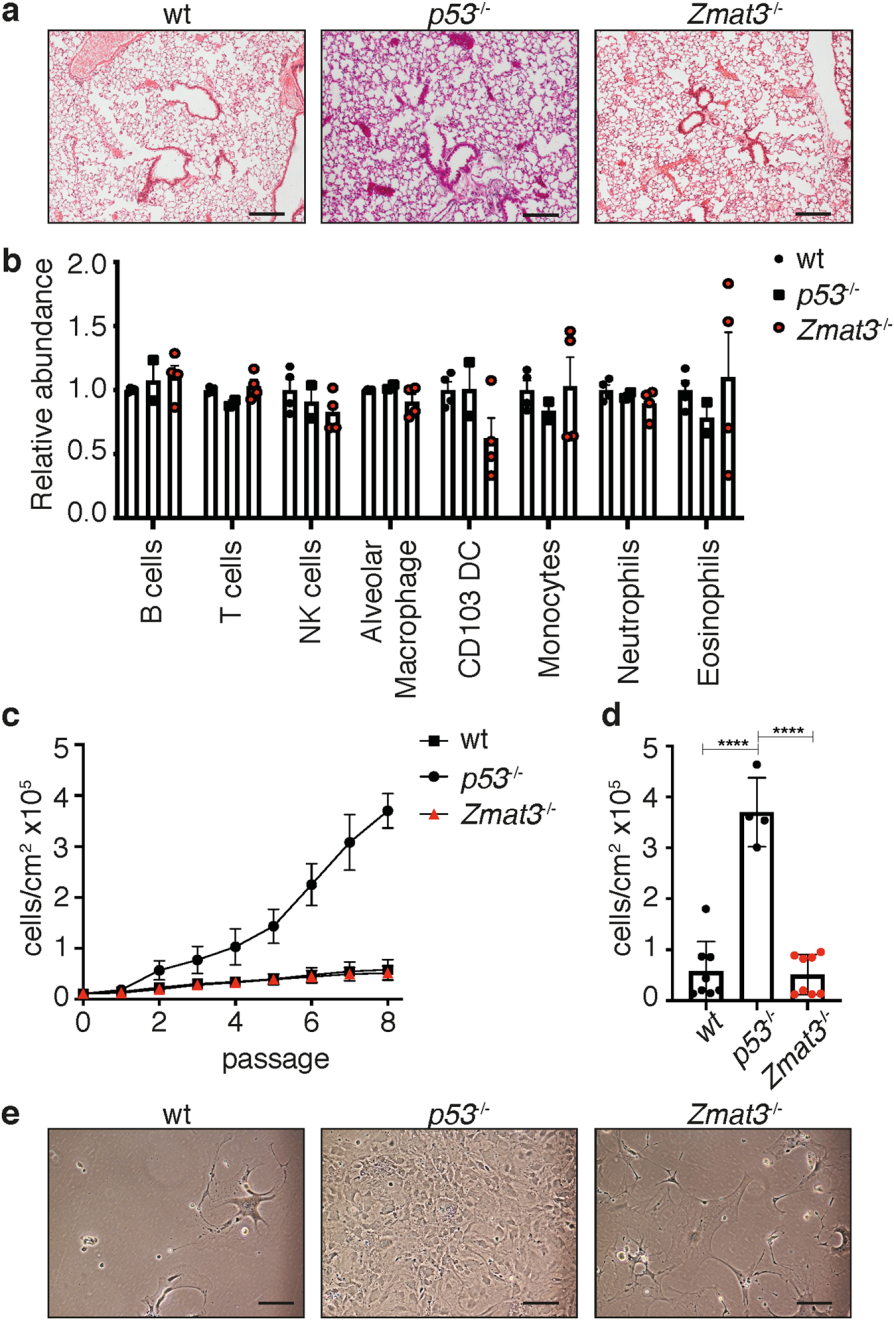
No effect of Zmat3 loss in the adult lung. **a** Representative hematoxylin and eosin (H&E) staining of lungs from *Zmat3*^−/^^−^, littermate *Zmat3*^+/+^ (wt) and *p53*^−/^^−^ control mice aged 16-18 weeks. Scale, 200 μm. **b** Flow cytometric analysis of the lymphoid and myeloid cells present in the lungs from *Zmat3*^−/−^ (*n* = 4), *p53*^−/^^−^ (*n* = 2) and wt (*n* = 4) mice. Immune cell populations are normalized to wt control mice. **c** Growth analysis of primary cell lines derived from the lung epithelium of wt (*n* = 8)*, Zmat3*^−/^^−^ (*n* = 8) and *p53*^−/^^−^ (*n* = 4) mice. Mean ± SEM. **d** Accumulative cell number following 8 passages of primary wt (*n* = 8)*, p53*^−^^/^^−^ (*n* = 4) and *Zmat3*^−/−^ (*n* = 8) cell lines. Mean ± SD, ****p < 0.0001. **e** Representative light microscope images of *Zmat3*^+/+^ (wt)*, p53*^−/−^ and *Zmat3*^−/^^−^ cell lines 8 passages following their derivation. Scale, 10 μm.

To further investigate the role of Zmat3 in oncogene driven tumorigenesis, we chose a lung adenocarcinoma (LUAD) model, in which p53 has been shown to play a critical tumor suppressive role^34^. In this model, activated *Kras*^G12D^ drives the development of lung adenomas, which progress to adenocarcinomas in the absence of p53^34^. First, we examined the expression of *Zmat3* in lungs isolated from wt and *Zmat3*^*−/*^^−^ mice, with or without activation of p53 by γ-irradiation. Indeed, *Zmat3* mRNA expression was markedly induced in wt lung tissue in response to DNA damage, but absent in the lung tissues from *Zmat3*^*−/−*^ mice (Supplementary Fig. 1a). To determine whether the loss of Zmat3 impacted normal development and homeostasis of the lung, we performed an in-depth examination of the cellular composition and immune microenvironment of the lung tissue from *Zmat3* deficient mice. As *Zmat3*^−/−^ mice are viable and healthy^14^, we aged *Zmat3*^−/^^−^ and *Zmat3*^+/+^ (wt) littermate controls to adulthood (16 to 18 weeks old) and evaluated the histopathology and cellularity (epithelial and immune) of the lungs of these mice. The lungs of *Zmat3*^−/^^−^ mice were histologically indistinguishable from the lungs of *p53*^−/^^−^ or wt control animals (Fig. 2a). Moreover, no differences in lung cellularity or weight were observed between *Zmat3*^−/−^ and controls (Supplementary Fig. 1b, c). Furthermore, the immune cell composition of the lungs was unchanged, with the levels of resident myeloid and lymphoid cell populations comparable between *Zmat3*^*−/*^^−^, *p53*^−/^^−^ and wt mice (Fig. 2b). To further investigate the role of Zmat3 in lung epithelium we generated primary cell cultures from *Zmat3*^−/−^, *p53*^−/^^−^ and wt adult lungs and examined the impact of the loss of Zmat3 on cell proliferation and cellular morphology in vitro. Cumulative cell counts (over 8 passages) revealed that *Zmat3*^−/−^ lung epithelial cells, similar to those from wt mice but unlike *p53*^−/−^ epithelial cells, failed to bypass replicative senescence (Fig. 2c–e). Consequently, similar to wt control cells, *Zmat3*^−/−^ cells underwent growth arrest exhibiting flattened appearance and multi-nucleation, characteristic of cells undergoing senescence (Fig. 2e). Collectively, these results show that the loss of Zmat3 has no impact on the development, homeostasis or immune cell composition of the murine lung and does not allow lung epithelial cells to evade cellular senescence in culture.

### Germline loss of *Zmat3* does not recapitulate *p53-*mediated acceleration of *Kras*^G12D^-induced tumorigenesis

**Fig. 3:**
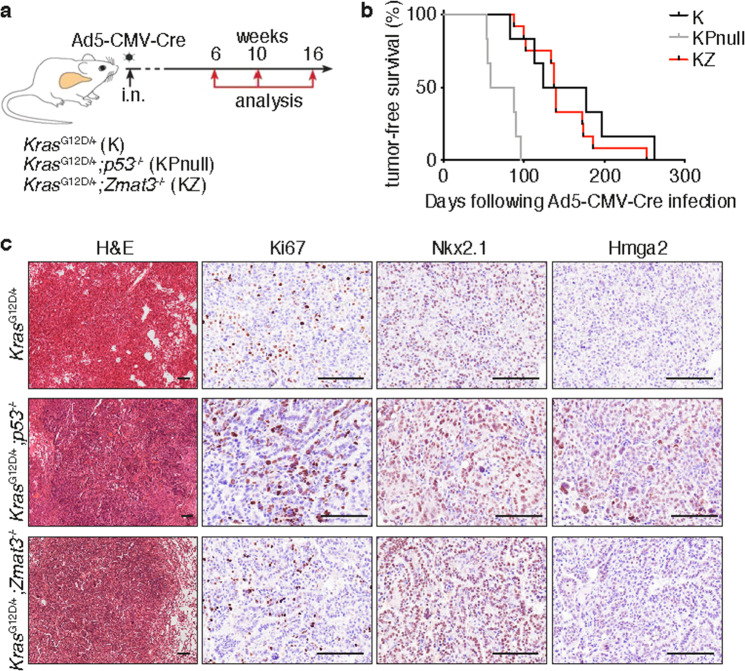
Loss of Zmat3 does not collaborate with mutant *Kras*^G12D^ in the development of lung adenocarcinoma. **a** Schematic of experimental design. *Kras*^LSL-G12D/+^ (K) mice^21^ were crossed with *p53*^−/^^−^ mice^37^ (KPnull) or *Zmat3*^−/^^−^ mice^14^ (KZ). **b** Kaplan–Meier tumor-free survival analysis of K (*n* = 6), KPnull (*n* = 6) and KZ (*n* = 12) mice following i.n. infection of Ad5-CMV-Cre virus. **c** Representative H&E staining and immunohistochemistry of Ki67, Nkx2.1/TTF-1 and Hmga2 in end-stage lung tumors from *Kras*^G12D^, *Kras*^G12D^;*p53*^−/^^−^ and *Kras*^G12D^;*Zmat3*^−/^^−^ mice. Scale, 100 μm.

Next, to functionally evaluate the significance of Zmat3 in *KRAS*-mutant LUAD, we crossed *Zmat3*^−/^^−^ mice with *Kras*^LSL-G12D/+^ mice^21^. Cohorts of *Kras*^LSL-G12D/+^ (hereafter; K), *Kras*^LSL-G12D/+^;*Zmat3*^−/−^ (hereafter; KZ) mice and *Kras*^LSL-G12D/+^;*p53*^−/^^−^ (hereafter; KPnull) mice were infected by intranasal inhalation with the ubiquitous Ad5-CMV-Cre adenovirus to recombine and activate mutant *Kras*^G12D^ expression in the respiratory epithelium^27^ (Fig. 3a). Consistent with previous reports utilizing a *p53* conditional allele^21^, germline loss of *p53* significantly accelerated lung cancer development in *Kras*^G12D^ mice (median survival K: 151 days vs KPnull: 72 days; *p* < 0.0001) (Fig. 3b). In contrast, loss of Zmat3 did not significantly alter the survival of *Kras*^G12D^ mice (median survival: KZ: 138 days versus K: 151 days) (Fig. 3b). Consistent with the animal survival data, we observed no differences in tumor histology between the lungs from the K vs KZ mice, nor the proliferation index, as determined by Ki67 staining (Fig. 3c, Supplementary Fig. 2a). The tumors from both K and KZ mice were defined as highly differentiated, reflected by high Nkx2.1 expression and low to absent expression of Hmga2 (Fig. 3c). In contrast, tumors from KPnull mice displayed increased numbers of Ki67 and Hmga2 positive nuclei, indicative of more aggressive and more poorly differentiated adenocarcinoma lesions (Fig. 3c, Supplementary Fig. 2b), consistent with the crucial tumor suppressive function of p53 in mutant *KRAS*-driven LUAD.

**Fig. 4:**
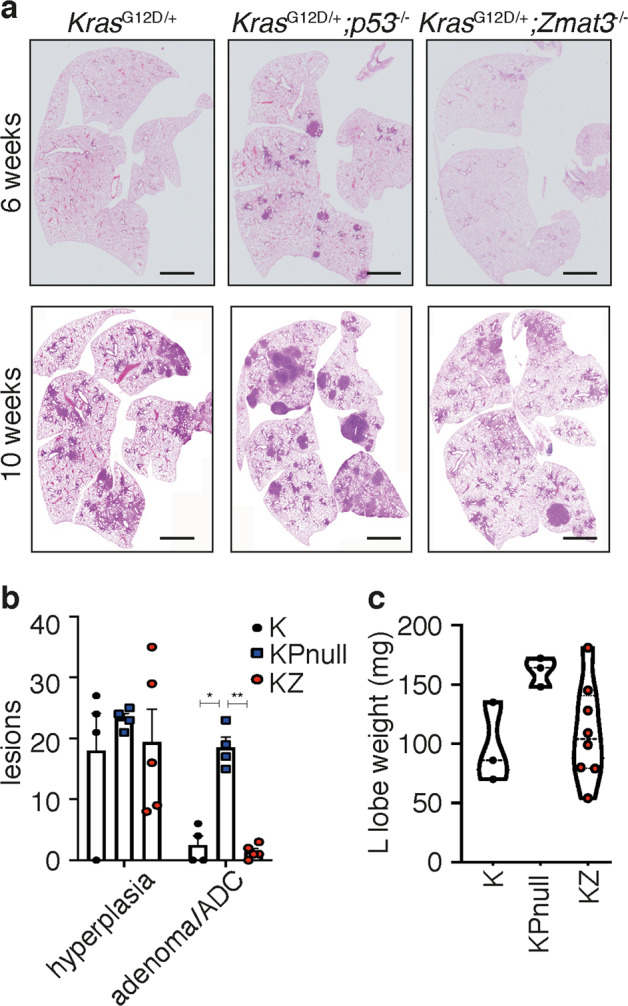
Zmat3 loss does not accelerate mutant *Kras*^G12D^-driven lung adenocarcinoma. **a** Representative low magnification H&E stained lungs of *Kras*^G12D/+^ (K), *Kras*^G12D/+^;*p53*^−/−^ (KPnull) and *Kras*^G12D/+^;*Zmat3*^−/−^ (KZ) mice 6- and 10-weeks following i.n. administration of Ad5-CMV-Cre. Scale, 1 mm. **b** Lesion classification in the lungs of K (*n* = 4), KPnull (*n* = 4) and KZ (*n* = 5) mice 10 weeks following i.n. Ad5-CMV-Cre infection. **c** Wet weight (mg) of the left lung lobe of K (*n* = 3), KPnull (*n* = 3) and KZ (*n* = 8) mice 6 weeks following i.n. Ad5-CMV-Cre infection. Violin plot indicates the median weight and distribution.

To evaluate whether Zmat3 loss impacts tumor initiation following oncogenic *Kras*^*G12D*^ activation, we performed a time course study, whereby K, KZ and KPnull mice were analyzed 6, 10 and 16 weeks following Ad5-CMV-Cre infection. While hyperplasic and adenomatous lesions were only apparent in lungs of KPnull mice 6 weeks following Ad5-CMV-Cre infection (Fig. 4a), sporadic lung lesions were observed in both K and KZ mice by 10 weeks, with further progression seen only in the lungs of KPnull mice (Fig. 4a). Quantification of the histopathology of lesions in KZ, KPnull and K mice 10 weeks following Ad5-CMV-Cre administration failed to reveal significant differences in tumor burden between K and KZ mice suggesting that *Zmat3* loss does not accelerate *Kras*^G12D^-induced tumorigenesis (Fig. 4b, Supplementary Fig. 2c). These findings were further verified in mice analyzed 16 weeks following Ad5-CMV-Cre infection, with no difference in tumor burden detected between K and KZ mice (Supplementary Fig. 2d).

Next, we assessed the tumor immune microenvironment using multiparametric flow cytometry. Consistent with *Kras*^G12D^-driven lung adenocarcinoma models, alveolar macrophages were abundant in the microenvironment of all genetic backgrounds tested, suggesting no alterations in this key feature of mutant *Kras*^G12D^-driven adenocarcinoma development due to the loss of *Zmat3*^35^. Furthermore, no differences were identified in the lymphoid or myeloid compartments between the K, KZ and KPnull cohorts (Supplementary Fig. 3a). Consistent with the presence of tumors in the lungs 10-weeks post Ad5-CMV-Cre administration, CD8^+^ T cells in the lungs of tumor-bearing mice were activated and displayed elevated PD-1 expression (Supplementary Fig. 3b). These findings show that loss of Zmat3 does not alter the key factors shown to impact the immune milieu under homeostatic conditions or in response to oncogene activation.

We have previously shown that lung weight is a robust readout of tumor burden in *Kras*^G12D^-driven GEMMs^35^. Interestingly, while lung weights were consistently in a relatively narrow range within the K (uniformly low lung weight) and KPnull cohorts (uniformly high lung weights), large variations in lung weights were observed between individual KZ mice (Fig. 4c). This may indicate some tumor suppressor function of Zmat3, perhaps dependent on oncogenic lesions cooperating with mutant *Kras*^G12D^ and the loss of Zmat3 that are present in some but not all KZ mice.

### *Zmat3* loss fails to rescue *Kras*^G12D^ lung adenocarcinoma cells from oncogene-induced senescence

**Fig. 5:**
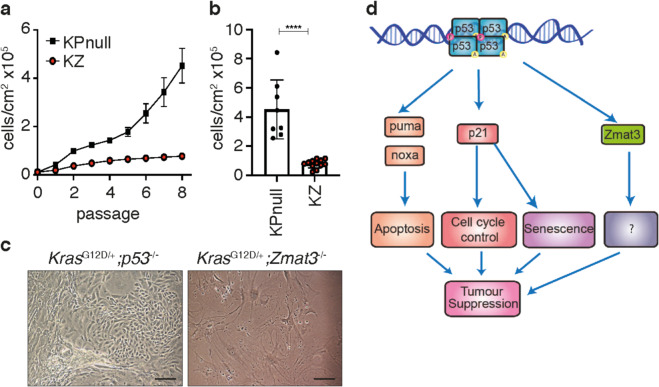
Zmat3 loss does not overcome mutant *Kras*^G12D^ induced cell senescence. **a** Growth analysis of primary cell lines derived from the lung epithelium of KPnull (*n* = 8) and KZ (*n* = 12) mice 10 weeks following i.n. Ad5-CMV-Cre infection. Mean ± SEM. **b** Accumulative cell number following 8 passages of primary KPnull (*n* = 8) and KZ (*n* = 12) cell lines. Mean ± SD, ***p < 0.0001. **c** Representative light microscope images of *Kras*^G12D^/*p53*^−/^^−^ (KPnull) and *Kras*^G12D^/*Zmat3*^−/^^−^ (KZ) cell lines 8 passages following their derivation. Scale, 10 μm. **d** Schematic representation of p53 target genes and their role in tumor suppression.

Loss of *p53* function prevents cell cycle arrest and enables evasion from cellular senescence in mutant *KRAS*-driven tumorigenesis. To assess whether loss of Zmat3 could promote evasion from oncogene-induced senescence (OIS) in *Kras*^G12D^ mutant lung epithelial cells, primary cell lines generated from KZ and KPnull lung tumors were assessed for proliferation over 8 passages. Consistent with previous findings^9^, cumulative cell counts demonstrated that while KPnull cells replicated indefinitely, and KZ cells underwent growth arrest that was evident after 4 passages (Fig. 5a–c). These results suggest that the loss of Zmat3, unlike the loss of *p53*, does not permit evasion from OIS in *Kras*^G12D^ driven lung tumorigenesis.

Taken together, we have demonstrated that in murine models of *Myc*-driven lymphoma and *Kras*^G12D^-driven LUAD, the germline loss of *Zmat3* did not have a considerable effect on the rate of onset, overall incidence or severity of tumor development, unlike *p53* loss. In humans, it has been shown that Zmat3 is expressed ubiquitously in all tissues and its deregulation has been implicated in various cancers, including leukemia as well as lung and breast cancer^15–17^. More importantly, our previous findings have shown that loss of Zmat3 causes leukemia only in overlap with concomitant loss of the p53 targets, *Puma* and *p21*^14^, that are critical for p53-mediated apoptosis and cell cycle arrest, respectively. Hence, it is possible that the tumor suppressive function of Zmat3 is more pronounced in other tissues and oncogene induced models. The understanding of the molecular mechanisms underlying p53s’ function in tumor suppression is constantly expanding. It is now clear that for p53-mediated tumor suppression other functions in the cell are critical in addition to induction of cell cycle arrest, apoptosis or senescence^4–9^. *Zmat3* is a p53 target gene that is involved in activation of cell cycle arrest or apoptosis indirectly, and as it regulates gene expression in a post-translational manner could modulate the p53 response towards other still unknown biological effector processes^15,36^. The inability of loss of *Zmat3* to cooperate with *c-MYC* and *KRAS* expression in tumorigenesis may be explained by the finding that Zmat3 regulates a large set of targets at the mRNA level^36^ and that its’ function is dependent on additional co-occurring mutations other than deregulated *c-MYC* expression, mutations in *KRAS* and cellular stress (Fig. 5d). Further studies will be required to investigate the function and biological significance of Zmat3 in epithelial and lymphoid tumorigenesis.

## Supplementary information

Supplementary Figure legend

Supplementary Figure S1

Supplementary Figure S2

Supplementary Figure S3
