# Supplementary material for: Consequences of Zmat3 loss in c-MYC- and mutant KRAS-driven tumorigenesis
Source: Cell Death Dis. 2020 Oct 20;11(10):877. doi: 10.1038/s41419-020-03066-9 (PMC7575595; doi:10.1038/s41419-020-03066-9)
Supplement: Supplementary file 1 — Supplementary Figure legend [file 41419_2020_3066_MOESM1_ESM.docx]

**SUPPLEMENTARY FIGURE LEGENDS**

**Supplementary Fig. 1.**

**a.** Lungs from wt and *Zmat3*^-/-^ mice were either exposed to 8 Gy irradiation or left untreated and harvested after 6 h. Fold change of *Zmat3* expression of γ-irradiated was standardized to the untreated lungs in wt (n=4) and *Zmat3*^-/-^ (n=4) mice. Data represent mean ± SEM. *Zmat3* mRNA was not detected in the lungs from *Zmat3*^-/-^ mice. **b.** Cell counts of whole left lung lobe digested to a single-cell suspension from wt (n=4), *p53*^-/-^ (n=4) and *Zmat3*^-/-^ (n=4) mice. Cell number normalized to the wt control on each experimental day. Mean ± SD**. c**. Left lobe wet weight (mg) from wt (n=4), *p53*^-/-^ (n=4) and *Zmat3*^-/-^ (n=4) mice. Mean ± SD**.**

**Supplementary Fig. 2.**

Quantification of **a.** Ki67^+^ cells and **b.** Hmga2^+^ cells relative to total tumor cells detected in representative *Kras*^G12D^ (K; n=2), *Kras*^G12D^;*p53*^-/-^ (KPnull; n=2) and *Kras*^G12D^;*Zmat3^-^*^/-^ (KZ; n=2) mice 10 weeks following Ad5-CMV-Cre inhalation and moribund mice (KM; Kaplan-Meir curve cohort) *Kras*^G12D^ (K; n=1), *Kras*^G12D^;*p53*^-/-^ (KPnull; n=2) and *Kras*^G12D^;*Zmat3^-^*^/-^ (KZ; n=2). **c.** Proportion of tumor area in the lungs of *Kras*^G12D^ (K; n=3), *Kras*^G12D^;*p53*^-/-^ (KPnull; n=4) and *Kras*^G12D^;*Zmat3*^-/-^ (KZ; n=5) mice 10 weeks following Ad5-CMV-Cre inhalation. *p= 0.013 Ordinary one-way ANOVA with Tukey’s multiple comparisons test. **d.** Representative H&E stained lung sections (left) and left lobe wet weight (right) of *Kras*^G12D/+^ (K; 6 weeks: n=3; 10 weeks: n=4; 16 weeks: n=4) and *Kras*^G12D/+^;*Zmat3*^-/-^ (KP; 6 weeks: n=8; 10 weeks: n=5; 16 weeks: n=5) mice 6, 10 and 16 weeks following i.n. administration of Ad5-CMV-Cre. Scale, 200 μm.

**Supplementary Fig. 3.**

**a.** Flow cytometric analysis of the lymphoid and myeloid cells present in the lungs of *Kras*^G12D/+^ (K; n=5), *Kras*^G12D/+^;*p53*^-/-^ (KPnull; n=4) and *Kras*^G12D/+^;*Zmat3*^-/-^ (KZ; n=4) mice. Immune cell populations are normalized to wt control mice (dotted line). **b.** Fluorescence intensity of PD-1 expression on CD8^+^ T cells isolated from the lungs of uninfected littermate (n=3), *Kras*^G12D/+^ (K; n=4), *Kras*^G12D/+^;*p53*^-/-^ (KPnull; n=4) and *Kras*^G12D/+^;*Zmat3*^-/-^ (KZ; n=4) mice. Expression relative to non-tumor bearing “normal” lung control (dotted line).
