# Supplementary figures and images for: Consequences of Zmat3 loss in c-MYC- and mutant KRAS-driven tumorigenesis
Source: Cell Death Dis. 2020 Oct 20;11(10):877. doi: 10.1038/s41419-020-03066-9 (PMC7575595; doi:10.1038/s41419-020-03066-9)

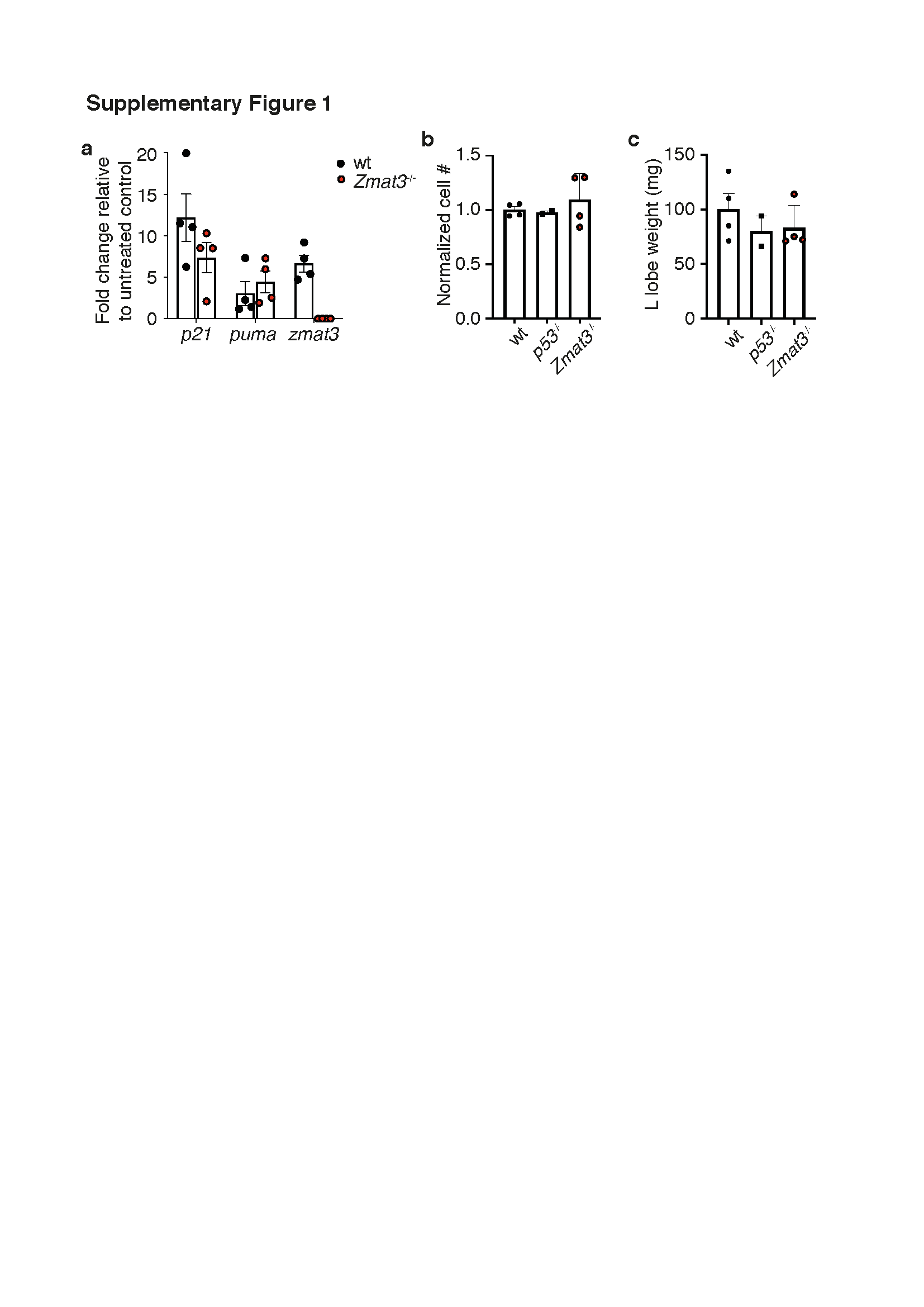

Supplement: Supplementary file 2 — Supplementary Figure S1 [file 41419_2020_3066_MOESM2_ESM.png]

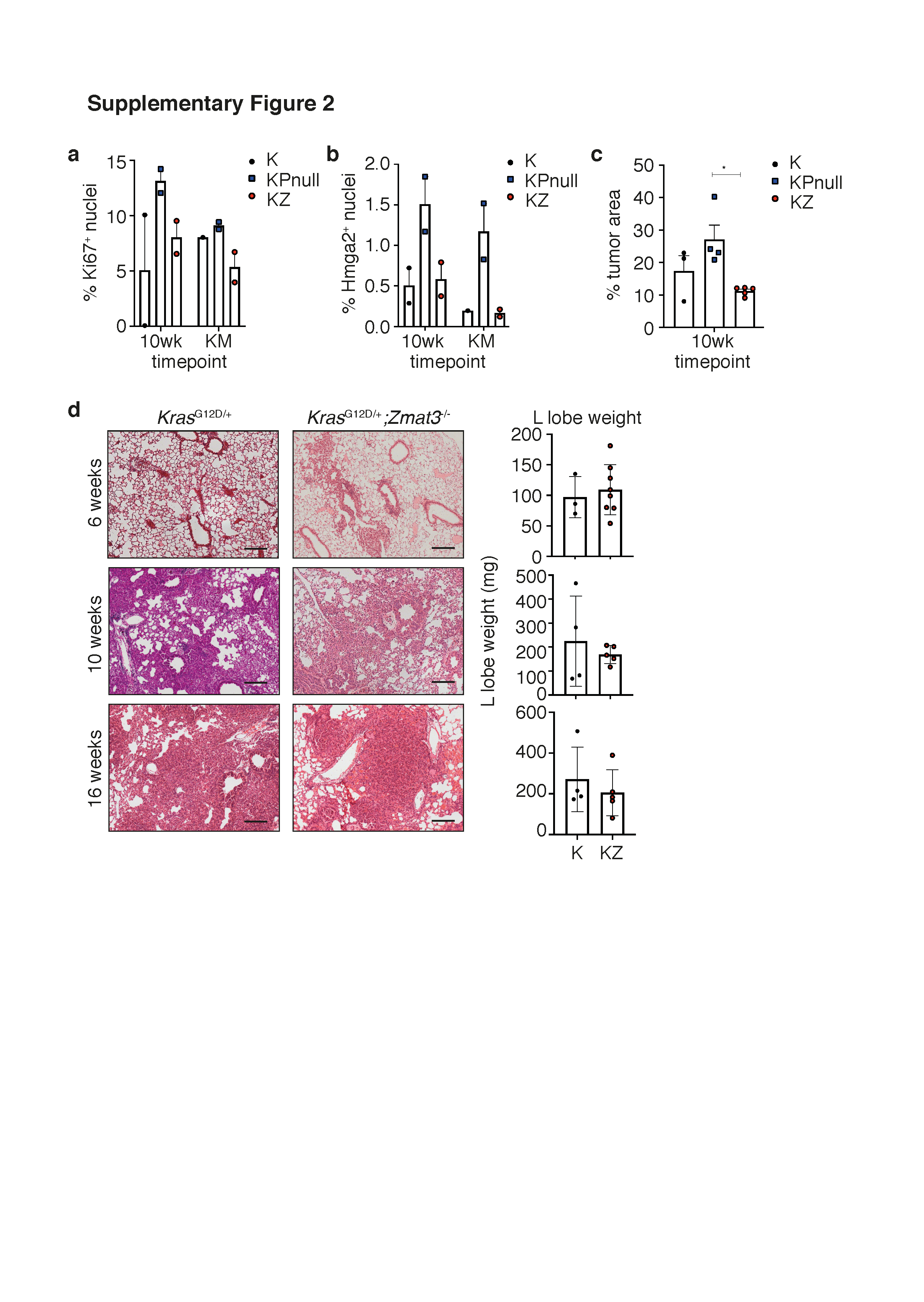

Supplement: Supplementary file 3 — Supplementary Figure S2 [file 41419_2020_3066_MOESM3_ESM.png]

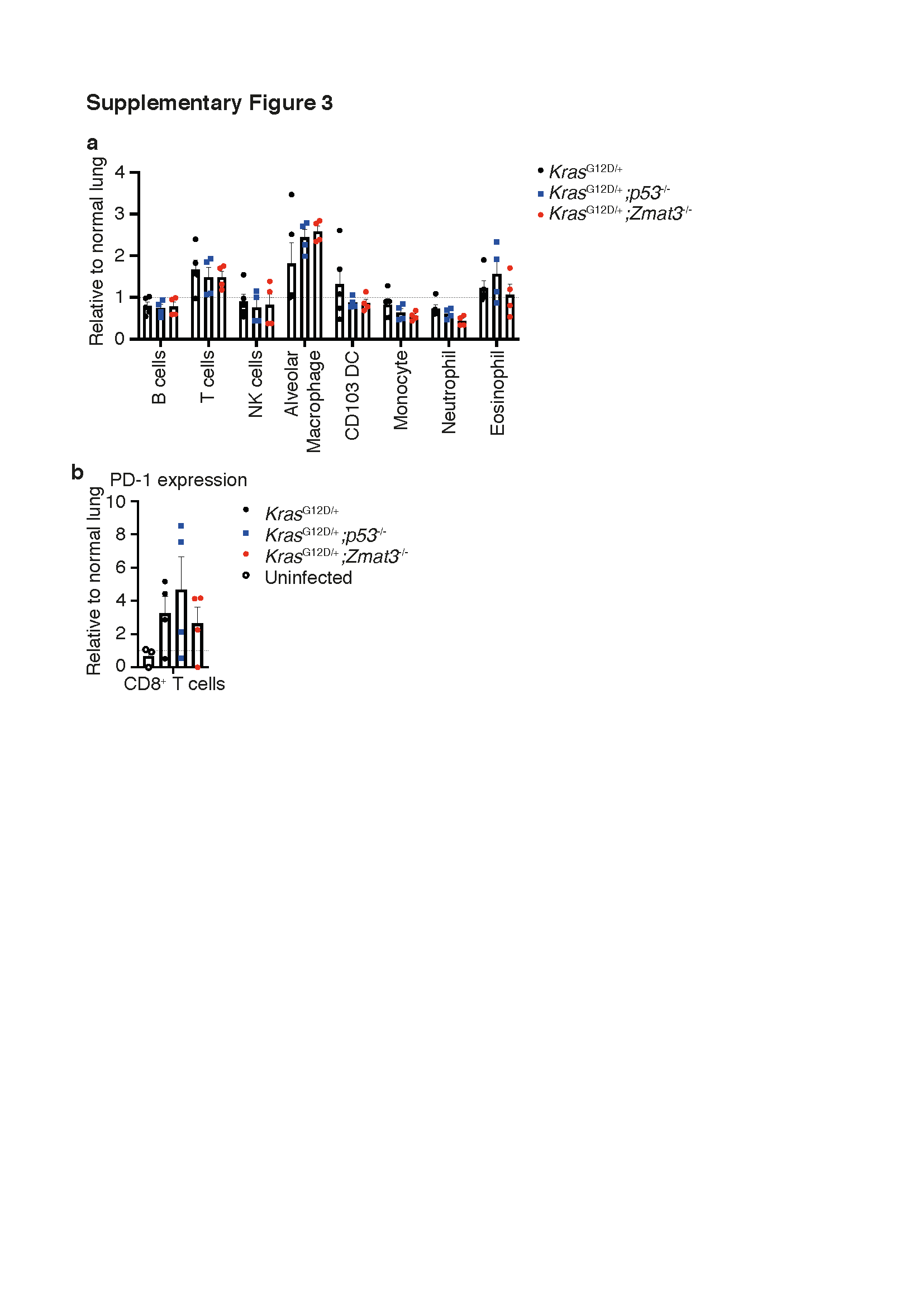

Supplement: Supplementary file 4 — Supplementary Figure S3 [file 41419_2020_3066_MOESM4_ESM.png]
